# Reversible Effects of Functional Mandibular Lateral Shift on Masticatory Muscles in Growing Rats

**DOI:** 10.3390/biomedicines11082126

**Published:** 2023-07-27

**Authors:** Hao Guan, Ikuo Yonemitsu, Yuhei Ikeda, Takashi Ono

**Affiliations:** Department of Orthodontic Science, Graduate School of Medical and Dental Sciences, Tokyo Medical and Dental University (TMDU), Tokyo 113-8549, Japan

**Keywords:** functional mandibular lateral shift, masseter muscle, temporalis muscle, myosin heavy chain, insulin-like growth factor-1, growth differentiation factor-8, early orthodontic treatment

## Abstract

In this study, we aimed to determine the effects of functional mandibular lateral shift (FMLS) on the muscle mass, fiber size, myosin heavy chain fiber type, and related gene expression in masticatory muscles (masseter and temporalis), as well as whether the baseline levels could be recovered after FMLS correction in growing rats. The FMLS appliance was placed to shift the mandible leftward by approximately 2 mm. After FMLS placement for 2 and 4 weeks, the muscles on the left side had significantly lower wet weight, mean cross-sectional area, and proportion of type IIa fibers than those on the right side or in the control groups (*p* < 0.05), with downregulation and upregulation of *IGF-1* and *GDF-8* gene expression, respectively (*p* < 0.05). Following 2 weeks devoted to recovery from FMLS, the muscle parameters in the recovery group were not significantly different to those of the control group, and *IGF-1* expression in the left-side muscles was enhanced and *GDF-8* expression was simultaneously suppressed. These findings indicate that the masticatory muscle changes induced via FMLS tend to revert to normal conditions if the intervention is eliminated at an early stage. Therefore, appropriate orthodontic treatment for FMLS during the growth period is advisable to prevent asymmetric alterations in masticatory muscles.

## 1. Introduction

Approximately 8–22% of orthodontic patients develop unilateral posterior crossbite (UPXB) [1,2,3], making it a common malocclusion in deciduous and early mixed dentitions [4]. Most cases of unilateral posterior crossbite are related to functional mandibular lateral shift (FMLS) [5], a condition in which the mandible laterally deviates to achieve maximal intercuspation due to premature contact between the teeth [6]. If left untreated for a prolonged period, FMLS can cause multiple pathological conditions, as well as functional crossbite [7,8,9].

Asymmetric masticatory muscle activity is one of potential side effects of FMLS [10,11,12,13,14,15]. Kiliaridis et al. [10] observed that the masseter muscle (MM) is significantly thinner on the crossbite side in UPXB individuals. Neuromuscular imbalance that involves masticatory muscle activity may contribute to FMLS [11,12]. Indeed, electromyography analysis has revealed that patients with UPXB exhibit asymmetric activities in masticatory muscles [13,14]. The masticatory muscle activity is reportedly greater on the contralateral side than the ipsilateral side [15]. It is generally accepted that early treatment of UPXB associated with FMLS may reduce the risk of potential side effects [16,17]. More specifically, early correction of UPXB can positively impact the MM and significantly reduce the occurrence of reverse chewing patterns [18]. Further, early treatment improves resistance to fatigue and contraction of the masticatory muscles on the crossbite side [19,20], suggesting that dysfunctional alterations can revert to normal conditions if this form of mechanical stimulation is eliminated at an early stage. Early orthodontic treatment, such as the removal of premature contacts using an expansion plate, can effectively prevent the evolution of these complications into permanent dentition.

Animal experiments have shown that lateral mandibular deviation induces morphological changes in the mandible and temporomandibular joint (TMJ), as well as substantial variations in cellular signaling [21,22,23,24]. A mandibular lateral shift can cause asymmetry regarding the location and size of the bilateral glenoid fossae in growing rats [21] and a propensity for dysfunctional remodeling of the ipsilateral condyle [22]. In addition, a thickened superficial layer of condylar cartilage, which is indicative of adaptive alterations in the TMJ, has been reported upon inducing FMLS in growing rats [23]. Further, a mandibular lateral shift can increase the expression of neuronal nitric oxide synthase and vascular endothelial growth factor in the MM, causing altered superoxide dismutase activity [25].

Although the effects of functional lateral shift on the mandible and temporomandibular joint have been extensively studied through conventional animal experiments, alterations in masticatory muscles have received less attention. Additionally, muscle adaptation related to the recovery of FMLS and the involved cellular mechanisms remains unclear. Therefore, we attempted to establish an FMLS rat model; investigate the changes in the muscle mass, fiber size, and myosin heavy chain (MHC)-type transition in the MM and temporalis muscle (TM) during FMLS; and determine whether these changes can be returned to the levels seen in the control group following recovery from FMLS to provide evidence to support early treatment of FMLS in growing rats. Moreover, gene expression of insulin-like growth factor-1 (*IGF-1*) and growth differentiation factor-8 (*GDF-8*) was examined to elucidate the possibly related cellular mechanisms.

## 2. Materials and Methods

### 2.1. Experimental Model of FMLS

**Figure 1:**
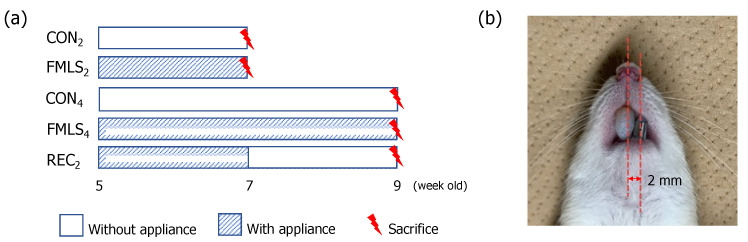
Experimental design. (**a**) Experimental groups and the study timeline: thirty-five 5-week-old male Wistar rats were randomly assigned to the following five groups (*n* = 7/group): groups with FMLS appliance placement for 2 (FMLS_2_) and 4 (FMLS_4_) weeks, groups without FMLS appliance placement for 2 (CON_2_) and 4 (CON_4_) weeks, and a group with placement of the FMLS appliance for the first 2 weeks and no appliance placement for the subsequent 2 weeks (REC_2_). (**b**) The FMLS appliance comprised guiding plates made of band material and light-cure resin. The appliance was attached to the maxillary and mandibular incisors to shift the mandible leftward by approximately 2 mm when the rats closed their mouths. FMLS, functional mandibular lateral shift.

The protocol used in this animal experiment was approved by the Animal Ethics Committee of Tokyo Medical and Dental University (No. A2021-116A). Thirty-five 5-week-old male Wistar rats were randomly assigned to five groups (*n* = 7/group): groups with FMLS appliance placement for 2 (FMLS_2_) or 4 (FMLS_4_) weeks, groups without FMLS appliance placement for 2 (CON_2_) or 4 (CON_4_) weeks, and a group with FMLS appliance placement for the first 2 weeks, followed by no appliance placement for the subsequent 2 weeks (REC_2_) (Figure 1a). 

Two rats were housed per cage in a climate-controlled environment that had a 12-hour light/dark cycle and given ad libitum access to powdered food (CLEA, Tokyo, Japan) and water. The rats were acclimatized to these conditions for the first 7 days, after which stage they received intraperitoneal injections of medetomidine, midazolam, and butorphanol tartrate. Under anesthesia, the FMLS_2_, FMLS_4_, and REC_2_ rats underwent placement of FMLS appliances. Although no appliances were placed for the CON_2_ and CON_4_ groups, the control rats were correspondingly anesthetized. 

The FMLS appliance, which was designed to unilaterally shift the mandible to the left by approximately 2 mm when the rats closed their mouths, comprised a pair of guiding plates located at the maxillary and mandibular incisors (Figure 1b). The guiding plate was made of band material (SHOHU INC., Kyoto, Japan) and covered with light-cure resin (TOMY International, Tokyo, Japan), as described previously [23]. The appliances were monitored to ensure an appropriate lateral shift and prevent mandibular protrusion or retrusion; the light-cure resin was checked daily and supplemented if necessary. In the REC_2_ group, the appliances were removed after 2 weeks of FMLS induction. 

### 2.2. Tissue Preparation

The procedures for euthanizing the rats were based on the standard CO_2_/O_2_ protocol at 7 and 9 weeks of age (*n* = 7/group). After euthanasia, the bilateral MM and TM were thoroughly and gently dissected at their attachment sites. The wet weight of the muscles was immediately measured, and the muscles were trimmed. For the quantitative reverse transcription polymerase chain reaction (RT-qPCR), approximately 80–90 mg of each muscle was collected, cut into smaller pieces of ≤0.5 cm in any single dimension, and stored at −80 °C until use in separate tubes filled with RNAlater™ Stabilization Solution (Invitrogen, Carlsbad, CA, USA), which was stored at 4 °C overnight in advance. Samples obtained from the muscle belly were submerged in an optimal cutting temperature-embedding compound (Sakura Finetek Japan Co., Ltd., Tokyo, Japan) and rapidly frozen in a supercooled bath of 2-methyl butane in liquid nitrogen. For each sample, transverse serial sections that had thicknesses of 10 μm were cut using a cryomicrotome (CM3500; Leica Microsystems, Nussloch, Germany). Sections were then stained with hematoxylin and eosin (H&E) to determine the cross-sectional area (CSA) of the fibers, and adenosine triphosphatase (ATPase) activity staining was performed to assess the MHC composition, along with immunohistochemical (IHC) staining for IGF-1 and GDF-8. 

### 2.3. ATPase Activity Staining

ATPase activity staining was performed according to the standard protocol outlined by the Neuromuscular Lab of the School of Medicine, Washington University. Non-fixation slides of snap-frozen muscles were first pre-incubated (in 5.0 mL barbital acetate solution, 10.0 mL 0.1 N hydrogen chloride, and 4.0 mL deionized water; adjusted to pH 4.6) for 5 min at room temperature and rinsed once with deionized water. Slides were then incubated in an adenosine triphosphate solution (0.18 M calcium chloride, 0.1 M sodium barbital and 60 mg adenosine triphosphate powder) of pH 9.4 for 25 min. Next, the slides were washed with 1% calcium chloride solution three times for a total of 10 min, incubated with 2% cobalt chloride for 10 min, washed with a 1:20 solution of 0.1-molarity sodium barbital three times, and rinsed with deionized water five times. Finally, the slides were incubated with a 2% (*v*/*v*) solution of ammonium sulfide for 20 s and rinsed in a fume hood with tap water for 5 min. The samples were then dehydrated in ascending concentrations of alcohol, cleared with at least two changes of xylene, and mounted. The stained sections were observed and photographed under an optical microscope (NIS-Element; Nikon, Tokyo, Japan), and the MHC fiber types were examined using ImageJ software (version 1.53) to determine the proportion of each type (National Institutes of Health, Bethesda, MD, USA). Subsequently, three non-overlapping high-magnification fields per muscle were randomly identified, typed, and numbered.

### 2.4. IHC Staining

Air-dried frozen sections were immersed in pre-cooled acetone (−20 °C) for 10 min. After allowing acetone to evaporate from the tissue sections for 20–30 min at room temperature, the sections were treated with 3% hydrogen peroxide for 15 min to block endogenous peroxidase activity. After rinsing in 1X phosphate-buffered saline (PBS) with 0.1% Tween-20 (ChemCruz^®^, Santa Cruz Biotechnology, CA, USA), with three changes that each lasted 5 min, the sections were incubated with 1.5% blocking serum (#sc-2023, ImmunoCruz^®^ goat ABC Staining System, Santa Cruz Biotechnology, CA, USA) for 1 h. After blotting excess blocking serum from the slides, the sections were separately incubated with goat anti-human IGF-1 primary antibody (concentration: 15 µg/mL; #AF291, R&D System, Minneapolis, MN, USA) and goat anti-human/mouse/rat GDF-8 primary antibody (concentration: 15 µg/mL; #AF788, R&D System, Minneapolis, MN, USA) in a humidified chamber at 4 °C overnight. The sections were then incubated with biotinylated secondary antibody for 1.5 h at room temperature, followed by 30 min of incubation with the AB enzyme reagent after washing three times with 0.1% PBS, with each wash lasting 5 min. 3,3-diaminobenzidine (#SK4105, ImmPACT DAB; Vector Laboratories, Burlingame, CA, USA) was used as a color-developing agent until the desired staining intensity was observed. Finally, the sections were counterstained with hematoxylin, dehydrated, and mounted. Three non-overlapping high-magnification fields for each sample were randomly selected, and positively immunostained areas were semi-quantified via IHC profiler plugins using ImageJ software.

### 2.5. RT-qPCR

Total RNA was isolated from the MM and TM samples stored in RNAlater ™ Stabilization Solution using TRIzol Reagent (Invitrogen, Carlsbad, CA, USA). Complementary DNA (cDNA) was synthesized from the isolated total RNA using reverse transcriptase and random primers from the PrimeScript RT Reagent Kit (#RR036; Takara, Tokyo, Japan). Real-time PCR analysis was performed using a 7500 Real-Time PCR system (Applied Biosystems, Foster City, CA, USA). Fluorescently labeled TaqMan Probes (#RR391, Probe qPCR Mix, Takara Biotechnology, Tokyo, Japan) and gene-specific primers were selected for real-time PCR amplification of messenger RNA (mRNA) of rat *IGF-1* (*Rn00710306_m1*), rat *GDF-8* (*Rn00569683_m1*), and rat *Gapdh* (*Rn01775763_g1*) (TaqMan Gene Expression Assay; Applied Biosystems, Foster City, CA, USA). *Gapdh* was used as the internal reference. Thermocycling was performed at 95 °C for 20 s, followed by 40 cycles of 95 °C for 1 s and 60 °C for 20 s. Gene expression levels were calculated using the Pfaffl method, being set as the relative quantification compared to the age-matched control group.

### 2.6. Statistical Analyses

Power analysis was performed using G*Power version 3.1.9.6 (Heinrich Heine University, Düsseldorf, Germany). The effect size was calculated using the data obtained from the pilot study. Next, the required sample size was determined (*n* = 6). Before comparison, the normal distribution of all variables was assessed using the Shapiro–Wilk test. After establishing normality, body weight and daily consumption of powdered food were determined using one-way analysis of variance, followed by Tukey’s multiple comparisons test. A paired *t*-test and Wilcoxon signed-rank test were performed for intragroup analyses between the deviation and non-deviation sides, and intergroup analyses were assessed using an unpaired *t-*test and a Mann–Whitney U-test. All statistical analyses were conducted using GraphPad Prism version 9 (GraphPad Software Inc., San Diego, CA, USA). Data are presented as mean ± standard deviation (*n* = 7). Statistical significance was set at *p* < 0.05 for all analyses.

## 3. Results

### 3.1. FMLS Rat Model

The FMLS appliances were kept in good condition in all groups during the experiment. No statistically significant differences in body weight were found between the control and experimental groups. During the first 1–2 days of appliance attachment, the rats attempted to remove the resin and band; however, this behavior ceased by the third day. The incisors grew during FMLS without extended eruption. We monitored the daily consumption of powdered food and water, and we confirmed that both were similar among the control and experimental groups.

### 3.2. Effect of FMLS on Wet Weight and CSA of Muscles

**Figure 2:**
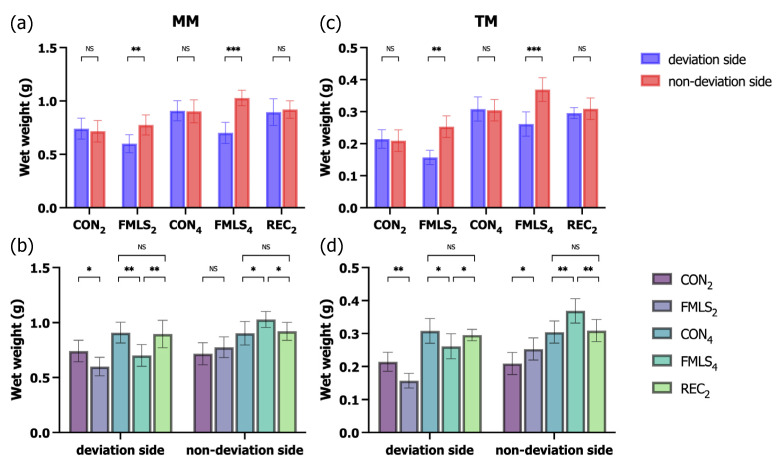
Effect of FMLS on muscle mass. (**a**) Wet weight of the MM (*n* = 7). (**b**) Wet weight of the MM (*n* = 7) on the deviation and non-deviation sides. (**c**) Wet weight of the TM (*n* = 7). (**d**) Wet weight of the TM (*n* = 7) on the deviation and non-deviation sides. FMLS, functional mandibular lateral shift; MM, masseter muscle; TM, temporalis muscle; NS, not significant. * *p* < 0.05, ** *p* < 0.01, *** *p* < 0.001.

The effects of FMLS on the wet weights of the MM and TM were first examined. In both the FMLS_2_ and FMLS_4_ groups, the wet weights of the MM and TM on the deviation side were significantly lower than those on the non-deviation side. Compared to those in the age-matched control groups, the wet weights of the MM and TM in the FMLS_2_ and FMLS_4_ groups decreased significantly on the deviation side and increased significantly on the non-deviation side. FMLS at 2 and 4 weeks resulted in atrophy of the MM and TM on the deviation side and muscle hypertrophy of the MM and TM on the non-deviation side (on the deviation side, CON_2_ vs. FMLS_2_, MM: 0.74 ± 0.09 g vs. 0.60 ± 0.08 g [*p* < 0.05], TM: 0.21 ± 0.03 g vs. 0.16 ± 0.02 g [*p* < 0.05]; CON_4_ vs. FMLS_4_, MM: 0.91 ± 0.09 g vs. 0.70 ± 0.10 g [*p* < 0.05], TM: 0.31 ± 0.04 g vs. 0.26 ± 0.04 g [*p* < 0.05]; on the non-deviation side, CON_2_ vs. FMLS_2_, MM: 0.72 ± 0.10 g vs. 0.78 ± 0.09 g [*p* > 0.05], TM: 0.21 ± 0.03 g vs. 0.25 ± 0.03 g [*p* < 0.05]; CON_4_ vs. FMLS_4_, MM: 0.90 ± 0.11 g vs. 1.03 ± 0.07 g [*p* < 0.05], TM: 0.30 ± 0.03 g vs. 0.37 ± 0.04 g [*p* < 0.05]). However, in the REC_2_ group, there was no significant difference between the deviation and non-deviation sides or that group and the CON_4_ group (Figure 2).

**Figure 3:**
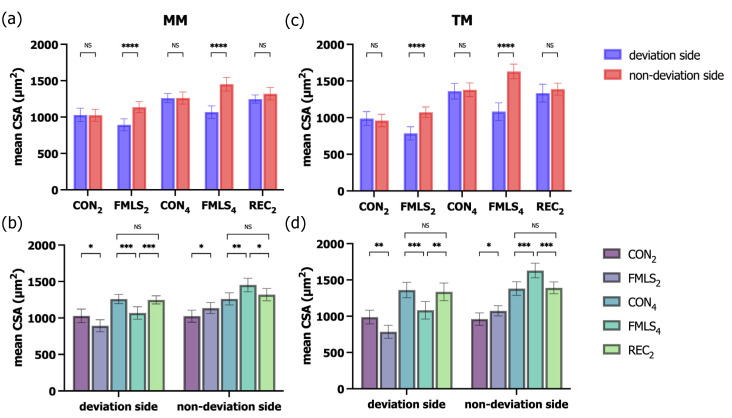
Effect of FMLS on fiber size. (**a**) Mean CSA of the MM (*n* = 7). (**b**) Mean CSA of the MM (*n* = 7) on the deviation and non-deviation sides. (**c**) Mean CSA of the TM (*n* = 7). (**d**) Mean CSA of the TM (*n* = 7) on the deviation and non-deviation sides. FMLS, functional mandibular lateral shift; CSA, cross-sectional area; MM, masseter muscle; TM, temporalis muscle; NS, not significant. * *p* < 0.05, ** *p* < 0.01, *** *p* < 0.001, **** *p* < 0.0001.

Muscle atrophy/hypertrophy was also examined in terms of the muscle CSA through H&E staining. The mean CSAs of the MM and TM on the deviation side in the FMLS_2_ and FMLS_4_ groups were significantly lower than those on each group’s non-deviation side. Compared to those of the CON_2_ and CON_4_ groups, the mean CSA increased significantly on the non-deviation side and decreased significantly on the deviation side in both the FMLS_2_ and FMLS_4_ groups. In the REC_2_ group, the mean CSA was similar on both sides, with no significant difference being found upon comparison with the mean CSA in the CON_4_ group (Figure 3).

### 3.3. Effect of FMLS on MHC Fiber Types

**Figure 4:**
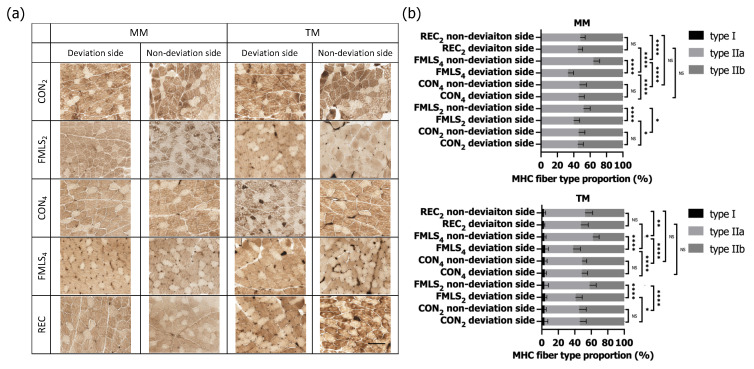
Effect of FMLS on MHC fiber types. (**a**) Representative images of ATPase activity staining of the MM and TM in each group. (**b**) MHC fiber-type proportions in the MM and TM. Scale bar represents 100 μm. ATPase, adenosine triphosphatase; MHC, myosin heavy chain; FMLS, functional mandibular lateral shift; MM, masseter muscle; TM, temporalis muscle; NS, not significant. * *p* < 0.05, ** *p* < 0.01, **** *p* < 0.0001.

For each side, three distinct fields per muscle sample were imaged at 200× magnification and used to determine the percentage of type I fibers, which were stained dark brown; type IIa fibers, which were stained light brown; and type IIb fibers, which were stained intermediate brown (Figure 4a). Type I fibers were observed in the TM, though they were not observed in the MM. In the MM, the proportions of type IIa fibers found on the deviation side in the FMLS_2_ and FMLS_4_ groups were significantly lower than those on the non-deviation side and in the CON_2_ and CON_4_ groups. Meanwhile, the proportion of type IIa fibers on the non-deviation side increased significantly, with concomitant reduction in type IIb fibers relative to the changes in the controls. In the REC_2_ group, compared to the FMLS_4_ group, the proportion of type IIa fibers on the deviation side significantly increased, reaching a level similar to that in the CON_4_ group. However, there were no significant differences in the proportions of type IIa and type IIb fibers between the two sides in the REC_2_ group. In the TM, in addition to the analogous alteration of type IIa fibers, no significant difference in the proportion of type I fibers was observed. Changes in the composition of the MHC isoforms in the MM and TM demonstrate that FMLS initiated a fiber-type slow-to-fast transition on the deviation side and a fast-to-slow transition on the non-deviation side. Additionally, the proportions of type IIa and type IIb fibers in the REC_2_ group tended to recover to the CON_4_ control level upon reversal of FMLS (Figure 4b).

### 3.4. IHC Staining for IGF-1 and GDF-8

**Figure 5:**
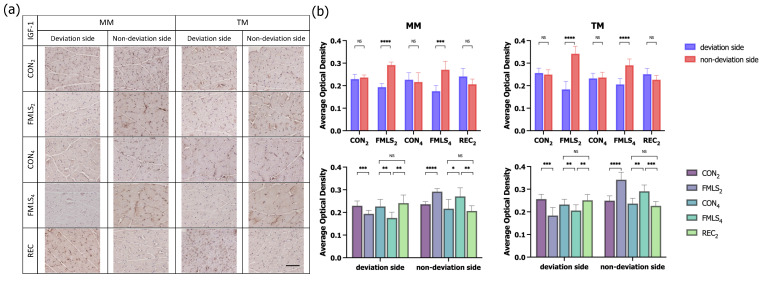
Immunohistochemical staining for IGF-1. (**a**) Representative images of IHC staining for IGF-1 in each group. (**b**) AOD of the area that was positively stained for IGF-1 in the MM and TM. Scale bar represents 100 μm. IGF-1, insulin-like growth factor-1; AOD, average optical density; MM, masseter muscle; TM, temporalis muscle; NS, not significant. * *p* < 0.05, ** *p* < 0.01, *** *p* < 0.001, **** *p* < 0.0001.

**Figure 6:**
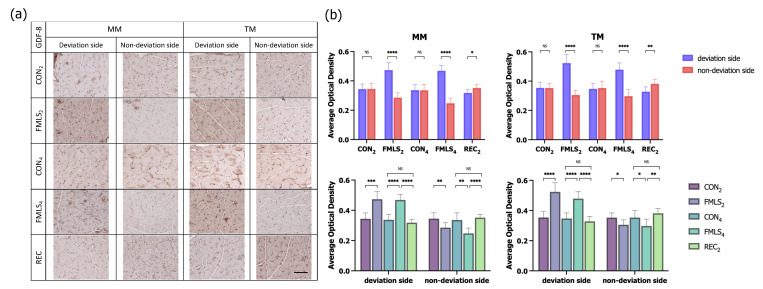
Immunohistochemical staining for GDF-8. (**a**) Representative images of IHC staining for GDF-8 in each group. (**b**) AOD of the area that was positively stained for GDF-8 in the MM and TM. Scale bar represents 100 μm. GDF-8, growth differentiation factor-8; AOD, average optical density; MM, masseter muscle; TM, temporalis muscle; NS, not significant. * *p* < 0.05, ** *p* < 0.01, *** *p* < 0.001, **** *p* < 0.0001.

To investigate the possible molecular signaling pathways involved in FMLS-induced muscle atrophy, hypertrophy, and fiber-type transition, the protein expression of IGF-1 and GDF-8, which are the main positive and negative regulators of muscle growth, was studied through IHC staining. The average optical density (AOD) of the positive area for each factor was semi-quantified to evaluate the level of protein expression, and three non-overlapping high-magnification fields per sample were randomly selected and evaluated. There was enhanced IGF-1 immunoreactivity in the MM and TM sections on the non-deviation side in the FMLS_2_ and FMLS_4_ groups. The AODs of IGF-1 in the MM and TM on the deviation side were significantly lower than those on the non-deviation side in the FMLS_2_ and FMLS_4_ groups. However, in the REC_2_ group, the AOD of IGF-1 on the deviation side was significantly greater than that in the FMLS_4_ group. In contrast, as one of the main negative regulators, GDF-8 immunoreactivity was enhanced in the MM and TM sections from the deviation side in the FMLS_2_ and FMLS_4_ groups, while it was enhanced from the non-deviation side in the REC_2_ group. The AODs of GDF-8 in the MM and TM on the non-deviation side in the FMLS_2_ and FMLS_4_ groups were significantly lower than those on the deviation side or in the control and REC_2_ groups. The protein expression of GDF-8 was upregulated on the deviation side, though it was downregulated on the non-deviation side, compared to the control groups, which is opposite to the trend of IGF-1 protein expression. Furthermore, the trend in the AOD of IGF-1 and GDF-8 in the REC_2_ group was opposite to that in the FMLS_4_ group (Figure 5 and Figure 6).

### 3.5. RT-qPCR for IGF-1 and GDF-8

**Figure 7:**
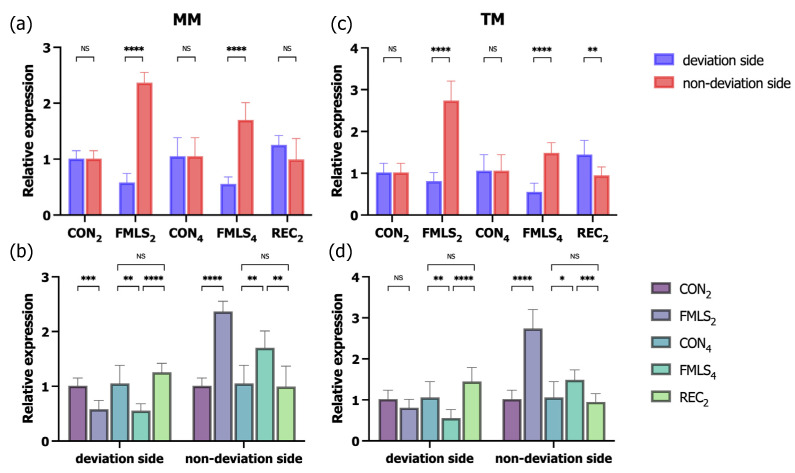
RT-qPCR for *IGF-1*. (**a**) Relative expression of *IGF-1* in the MM (*n* = 7). (**b**) Relative expression of *IGF-1* in the MM (*n* = 7) on the deviation and non-deviation sides. (**c**) Relative expression of *IGF-1* in the TM (*n* = 7). (**d**) Relative expression of *IGF-1* in the TM (*n* = 7) on the deviation and non-deviation sides. RT-qPCR, reverse transcription polymerase chain reaction; *IGF-1*, insulin-like growth factor-1; MM, masseter muscle; TM, temporalis muscle; NS, not significant. * *p* < 0.05, ** *p* < 0.01, *** *p* < 0.001, **** *p* < 0.0001.

**Figure 8:**
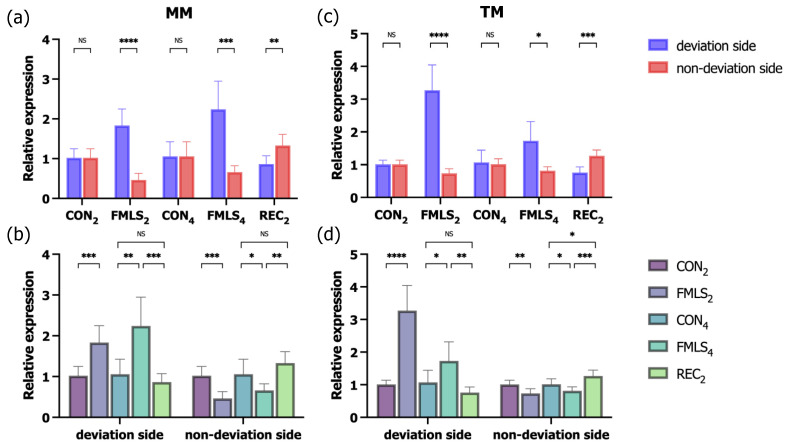
RT-qPCR for *GDF-8*. (**a**) Relative expression of *GDF-8* in the MM (*n* = 7). (**b**) Relative expression of *GDF-8* in the MM (*n* = 7) on the deviation and non-deviation sides. (**c**) Relative expression of *GDF-8* in the TM (*n* = 7). (**d**) Relative expression of *GDF-8* in the TM (*n* = 7) on the deviation and non-deviation sides. RT-qPCR, reverse transcription polymerase chain reaction; *GDF-8*, growth differentiation factor-8; MM, masseter muscle; TM, temporalis muscle; NS, not significant. * *p* < 0.05, ** *p* < 0.01, *** *p* < 0.001, **** *p* < 0.0001.

To more accurately determine the effect of FMLS on the regulation of related genes, the corresponding mRNA expression of *IGF-1* and *GDF-8* was measured using RT-qPCR. In the FMLS_2_ and FMLS_4_ groups, *IGF-1* mRNA expression on the non-deviation side was significantly upregulated 2.4- and 1.7-fold (MM) and 2.7- and 1.5-fold (TM), respectively, compared to that in the control group. In the FMLS_4_ group, *IGF-1* mRNA expression on the deviation side was significantly downregulated 0.5-fold in both the MM and TM. Consistent with the IHC findings, *IGF-1* mRNA expression in the TM on the deviation side in the REC_2_ group increased 1.4-fold, and it was significantly higher than that on the non-deviation side. Following 2- and 4-week FMLS, *GDF-8* mRNA expression on the deviation side was significantly enhanced 1.8- and 2.2-fold, respectively, in the MM and 3.3- and 1.7-fold, respectively, in the TM. Conversely, on the non-deviation side in the FMLS_2_ and FMLS_4_ groups, *GDF-8* mRNA expression was significantly downregulated, falling 0.5- and 0.7-fold, respectively, in the MM and 0.7- and 0.8-fold, respectively, in the TM. Following a 2-week recovery period after removing the FMLS appliances, *GDF-8* mRNA expression in both the ipsilateral MM and TM in the REC_2_ group decreased to a level equal to that in the CON_4_ group, though it significantly increased in the contralateral TM compared to the control group (Figure 7 and Figure 8).

## 4. Discussion

In this study, an appliance was designed to induce FMLS in growing rats by enabling the mandible to laterally deviate at an optical distance of approximately 2 mm, which has been verified in other studies regarding its effectiveness [23,26,27,28,29]. The FMLS appliance can be stably maintained for a considerable period (≥4 weeks) and harmlessly withdrawn to allow tissue recovery. In the present study, no significant difference in body weight or daily food consumption was observed among the groups, suggesting that the 2-millimeter mandibular shift did not cause nutritional concern. The experimental period was set to include the peak growth of masticatory muscles from a histological perspective, which extended from early puberty (5 weeks of age) to young adulthood (9 weeks of age) [30,31]. Hence, this experimental model enabled the evaluation of FMLS’ effects on the MM and TM during the rapid growth phase.

Muscle atrophy and hypertrophy, in terms of muscle mass and fiber size, occur in response to mechanical stimuli, as shown in past studies that used several in vivo and in vitro experimental models [32,33,34]. This study demonstrated the effects of FMLS on the two main masticatory muscles, i.e., MM and TM [35]. We observed remarkable changes in the wet weight and fiber CSA of the muscles on both sides in the experimental groups. With a significant difference between the deviation and non-deviation sides of the FMLS compared to age-matched control groups, FMLS tended to cause muscle atrophy on the deviation side and muscle hypertrophy on the non-deviation side. The REC_2_ group showed that early recovery from FMLS suppressed the FMLS-triggered growth and reduction in muscle mass and fiber size. As such, this study has shown, for the first time using an animal model, the effects of FMLS on masticatory muscles after recovery. 

Mechanical stimulus is a primary variable that regulates the growth and development of skeletal muscles [36,37,38]. In the present study, FMLS may have promoted an active adaptive response on both sides by inducing mechanical stress and environmental stimuli. A previous study of condylar modifications in an FMLS rat model showed asymmetric morphological changes in the condyle, indicating that the contralateral side experienced larger loads (tensile forces and shear stress) [23,39]. This notion is further supported by the current findings, as the contralateral muscles in the FMLS groups exhibited a significant increase in wet weight and fiber CSA compared to those in the control groups, whereas the ipsilateral MM and TM maintained a smaller size. The contralateral side was loaded with more compressive forces via FMLS when the rats closed their mouths, which was similar to those forces experienced during incising. Moreover, considering the asymmetric mandibular ramus height observed in previous FMLS animal models [40], together with the fact that different distances between muscle attachment sites lead to the stimulation or inhibition of muscle growth [41], we postulate that a longer ramus on the non-deviation side has a positive impact on muscle hypertrophy. Thus, the masticatory muscles alter and adapt to asymmetric changes in the mandible and condylar cartilage during FMLS. Muscle atrophy and lower contraction forces on the deviation side, which act as manifestations of the self-adaptability of living organisms, hinder the progression of the lateral shift and its side effects.

The fiber compositions of skeletal muscles are prenatally pre-programmed, though they can be altered and refined based on post-natal environmental factors and functional requirements [42,43]. The presence of multiple MHC isoforms is the key to understanding the extraordinary flexibility of skeletal muscles and their ability to adapt to a wide range of functional requirements [44,45]. This observation also justifies ATPase activity staining being performed in the present study to evaluate masticatory muscle adaptation during FMLS. We observed a tendency for a fast-to-slow transition of the masticatory muscle fibers on the non-deviation side in response to FMLS. With a lower muscle mass and smaller CSA, fibers on the deviation side had fewer type IIa oxidative fibers than those on the non-deviation side and in the control groups. The reduction in oxidative type IIa fibers indicates that the animals less frequently utilized their muscles [46]. In agreement with the finding that the ipsilateral muscle mass and fiber size decreased compared to the control level, the idea was further supported by the fact that masticatory muscles on the deviation side experienced reduced mechanical stimulus and eventually developed lower muscle activity.

To better elucidate the potentially related mechanisms, IHC staining and RT-qPCR were performed to explore the effects of FMLS on *IGF-1* and *GDF-8*, which are involved in primary muscle hypertrophic and atrophic signaling, respectively [47,48]. The present results indicate that FMLS-induced muscle hypertrophy may be associated with augmented *IGF-1* expression and inhibited *GDF-8* expression in the MM and TM on the non-deviation side. This finding is consistent with the results of a previous in vitro investigation that reported an association between enhanced *IGF-1* gene expression and a marked increase in muscle mass and fast-to-slow fiber-type transition [49]. The present results also corroborate the findings of previous studies, which suggested that *GDF-8* and *IGF-1* are negative and positive regulators of growth and proliferation in rat MM, respectively. Therefore, by combining the findings of the present and previous studies, it can be logically assumed that the asymmetric bilateral masticatory muscle changes in the experimental groups may be correlated with *IGF-1* and *GDF-8* regulation. More importantly, the early removal of the FMLS appliance in the REC_2_ group appeared to attenuate the FMLS-induced effects on *IGF-1* and *GDF-8* expression, which may account for the reversible changes and potential adaptation of the MM and TM. However, significant differences in *GDF-8* expression between the deviation and non-deviation sides in the MM and TM and *IGF-1* expression in the TM in the REC_2_ group were retained. Compared to the CON_4_ group, *GDF-8* expression on the non-deviation side was significantly enhanced in the REC_2_ group, as it appears that even after the removal of the FMLS appliance for 2 weeks, the normal level of related gene expression was not immediately reached. Studies using a novel group with an extended period of recovery may provide further evidence in this regard.

From a clinical perspective, functional adaptation and soft tissue regeneration after orthodontic treatment are important components of healing and rehabilitation. Indeed, certain parallels exist in the histopathological characteristics of TMJ between rats and humans, including the articular disc and capsule and the thick MM [50]. Based on these similarities, researchers have attempted to demonstrate the importance of early orthodontic treatment from different perspectives. Wattanachai et al. [26] and Kure-Hattori et al. [27] observed asymmetric changes in the proliferation and expression of lubricin in the bilateral condylar cartilage. Further, Yang et al. [23], Guo et al. [28], and Sato et al. [29] suggested that early treatment is necessary for normal growth and morphological corrections of TMJ, with this finding being indicative of mounting evidence that untreated crossbites can cause permanent developmental alterations, making it crucial that patients received treatment at an early age. Our study, which used the rat FMLS model for the first time to examine the changes in masticatory muscles, further demonstrates that FMLS can initiate adaptive changes in the MM and TM, while early elimination of this mechanical stimulus can aid reversion to normal conditions. 

Overall, it is reasonable to assume that skeletal, joint, and muscle adaptations due to FMLS occur at an early developmental stage. Once these reversible adaptations become permanent in adulthood, a combined orthodontic and surgical approach may be necessary to achieve reversal [5,6]. Thus, appropriate orthodontic treatment should be administered early in the growth phase to realize the potential advantages of correcting malocclusion related to FMLS. 

## 5. Conclusions

The present findings demonstrate that FMLS-induced changes in the MM and TM are reversible and tend to return to the baseline condition upon early elimination of FMLS during the growth period. This finding emphasizes the importance of early orthodontic treatment, as it helps patients to achieve balanced development of masticatory muscles.

## Data Availability

Not applicable.
